# Response of chemical and biochemical soil properties to the spreading of biochar-based treated olive mill wastewater

**DOI:** 10.1016/j.heliyon.2024.e31157

**Published:** 2024-05-14

**Authors:** Giuseppe Di Rauso Simeone, Giuseppina Scala, Marcello Scarpato, Maria A. Rao

**Affiliations:** Department of Agricultural Sciences, University of Naples Federico II, via Università 100, 80055 Portici, Italy

**Keywords:** Total phenols, Phytotoxicity, Soil organic amendment, Microbial biomass, Soil enzymes, Waste reuse

## Abstract

Despite the polluting potential olive mill wastewater (OMW) can be a useful source of nutrients and organic compounds to improve soil properties. The aim of this paper was to verify if biochar-based treatment of OMW could be an efficient method to contrast the richness in phenolic compounds and phytotoxicity of OMW making it more suitable. for soil amendment. In this study poplar biochar (BP) was more effective than conifer biochar (BC) in terms of adsorbing phenols and reducing phytotoxicity at different biochar rates (5 and 10 %). In soil amendment BP-treated OMW induced an increase of organic carbon by approximately 15 % and notably BP10 treated OMW enhanced available phosphorous by 25 % after 30 days of incubation. In soil amended with 10 % BP-treated OMW microbial biomass and enzymatic activities were significantly enhanced after 30 and 90 days, with no effect on cress seed germination. Therefore, biochar based-treatment could be cost-effective and able to facilitate the long-term management of OMW in terms of storage and disposal.

## Highlights

• Poplar biochar at 10 % rate was able to reduce phytotoxicity of OMW especially in term of seed germination (100 %) and phenolic compounds content up to 28 % of the initial concentration after 60 d.
• The soil amendment with biochar treated OMW increased the OC (+15 %) and stimulated also four-fold MB-C by after 30 d-incubation time.
• Soil amended with biochar treated OMW showed no phytotoxicity on cress seeds.

## Graphical abstract

**Figure:**
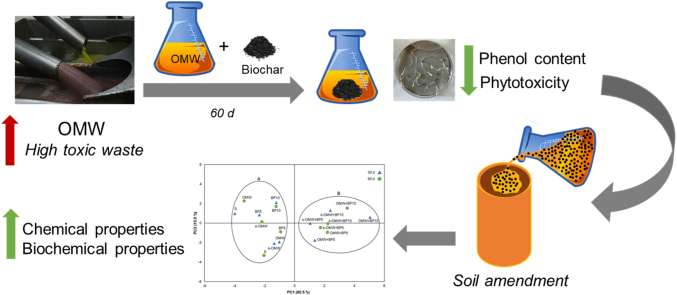

## 1 Introduction

In olive mills, the three-phase process produces wastewater (OMW) in huge amounts in a short period of a few months (October–February) [1]. OMW due to its toxicity and volumes accumulated per year (exceeding 30 million tons) generates serious concerns in the Mediterranean environment [1]. To avoid the possible toxicity for crops and soil microorganisms and the OMW leaching to superficial and groundwater and therefore their contamination, the disposal of OMW on soil as an organic amendment can occur only following a specific regulation. Moreover, a contained use of OMW in the organic amendment on agricultural soils could represent a sustainable strategy able to lead to a significant soil fertility improvement [[2], [3], [4], [5]]. Concerns for plant and soil microbial communities deriving from OMW arise because it is very rich in organic matter but particularly in phenolic compounds, fatty acid salts, and recalcitrant molecules such as lignins and tannins [1,[6], [7], [8]]. As a consequence, the chemical oxygen demand (COD) and the biological oxygen demand (BOD) of OMW reach very high levels such as around 15,000–135,000 mg L^−1^ and around 37,000–318,000 mg L^−1^, respectively [1,[7], [8], [9]]. On the other side, polyphenols present in high concentrations in OMW (up to 10 g L^−1^) can represent great added-value compounds for pharmaceutical, cosmetic, and health industries, including food preservation and packaging [10].

Pre-treatments able to reduce the OMW toxicity allow its disposal limiting the secondary effects especially when a huge amount of OMW must be managed [11]. As suggested by Cassano et al. [12] ultrafiltration using different membrane types proves to be effective in the OMW treatment. Among tested membranes, those made with polyethersulfone are more able than cellulose-based membranes to remove phenolic molecules from treated OMW. As an alternative method, direct contact membrane distillation (DCMD) with polytetrafluoroethylene membranes seems working well [13]. Nevertheless, the phenomenon of membrane saturation usually occurring in filtration systems reduces the efficiency of the process and increases their cost at the same time. Therefore, new proposals of pre-treatments are necessary to handle OMW to diminish membrane fouling, raise the efficiency of the filtration process keeping its costs low [9,12].

Among OMW treatments, biological methods were considered less expensive and environmentally friendly than physical and chemical ones [[14], [15], [16], [17], [18], [19]]. For example, in our latest research work, we have successfully applied an anaerobic digestion process on OMW to produce biogas [18]. Biological solutions could be a valid option although the possible presence of inhibitors or toxic chemicals, such as polyphenols and lipids, makes this treatment not easily feasible [1,18,20]. To reduce OMW phenolic content the use of biochar could be an economical and eco-friendly alternative. Kasozi et al. [20] showed that biochar could be used as an efficient catechol sorbent. Yakout [21] found that biochar prepared from rice straw at various pyrolysis temperatures was particularly effective in removing phenols from industrial effluent. Micoli et al. [18] found that biochar addition before or during the anaerobic digestion process reduced the phenolic compounds, COD, and phytotoxicity of OMW.

Biochar is a carbonaceous material produced through pyrolysis of sustainably gained biomass using clean technology [22,23]. It is rich in aromatic groups and minerals [22] and has a great surface area and microporosity, which make biochar able to adsorb organic and inorganic pollutants [[24], [25], [26], [27]] and therefore, a valid tool in environmental remediation [28,29]. There are two factors affecting biochar's adsorption capabilities: the pyrolysis temperature and the original biomass [27,30]. By raising the pyrolysis temperature (from 250 to 650 °C), biochar made from oak, pine, and grass showed increased catechol adsorption [27], but, on the other side, the entity of catechol adsorption depended on the kind of biomass (pine, oak, grass), as well as the size of the biochar particles (i.e. coarse, fine) [27]. If biochar can adsorb phenols and other more complex contaminants in simplified contaminated systems [7,21,27], we can hypothesize biochar could be efficient in removing phenols in a complex system like OMW, in which phenols are one of the principal causes of toxicity. A process providing biochar-based treatment of OMW before spreading it in agricultural soils could make this wastewater a useful organic amendment for the enhancement of soil physical, chemical, and biological characteristics, and as a consequence an increase in soil fertility [31].

Therefore, this work aimed to evaluate the ability of two different biochar produced from poplar and conifer wood to remove/retain phenol compounds from OMW thus reducing their toxicity. Once treated with biochar, OMW were used as organic fertilizer for soil. The effect of biochar-treated OMW on the chemical and biochemical properties of agricultural soil was also investigated to evaluate any soil fertility enhancement. Findings of this paper will facilitate waste management of olive oil mill plants, producing big volumes of OMW in a short time, ma king OMW a useful resource to improve soil quality through a circular economy approach.

## 2 Material and methods

### 2.1 Biochar characteristic

Chemical properties such as pH, elemental composition, content of ash, and metals of poplar (BP) and conifer (BC) biochar were measured by De Pasquale et al. [32]. The surface area measured by BET (Brunauer–Emmett–Teller) analysis of BP and BC was 76.88 m^2^ g^−1^ and 114.67 m^2^ g^−1^, respectively [32]. The FT-IR analysis demonstrated BP and BC did not differ in terms of functional groups [22].

### 2.2 Soil and olive mill wastewater sampling strategies

The soil used in this experiment was collected from citrus orchard located in Portici in the South of Italy (40°49′11″ N, 14°20′28″ E) from the 10–20 cm soil layer, sieved at 2 mm, and immediately used in the lab-scale experiment. A suitable amount of soil was air-dried at room temperature and analyses to measure pH, electrical conductivity (EC), OC, N, P, and soil texture were performed according to the literature [[33], [34], [35]]. Briefly, the pH and EC were measured in a soil/water 1:2,5 and 1:5 (w/v) mixture, respectively. Organic C (OC) content was assayed by dichromate oxidation titration method, while total N was determined by the Kjeldahl method. Available phosphorous was determined following the bicarbonate extraction process.

Another suitable amount was stored at 4 °C for biochemical analyses described in the paragraph 2.8 [36].

OMW was collected from three phase olive mill extraction plant located in San Prisco (Caserta) in the South of Italy and stored at −20 °C until use. The detailed process involving OMW from the collection to soil amendment is described in Fig. S1.

### 2.3 Characterization of OMW

The pH and EC (mS cm^−1^) of OMW samples were measured using a pH meter (Hanna Instruments, Hi 9017 electrode CW711) and conductivity meter (Hanna Instruments, Hi 8733), respectively. Total phosphorus, COD, and BOD_5_ were measured according to the APAT methods [37].

Phenolic compounds were extracted following on the method reported by Brenes et al. [38]. Briefly, in a 50 mL tube 20 mL of distilled water was added to 20 mL of OMW and 5 mg of sodium metabisulfite (400 mg L^−1^) to prevent phenol oxidation. Syringic acid (1 mL of 200 mg L^−1^ solution) was added as an internal standard. The solution was washed three times with 15 mL of *n*-hexane to remove the lipidic fraction. All solutions were collected in a separatory funnel and each sample was washed five times with 20 mL of ethyl acetate. The extract was concentrated by evaporation under vacuum (LABOROTA 4000, Heidolph) and dissolved in 2 mL of methanol.

The total phenolic compounds of extracts were measured by using the Folin-Ciocalteu reagent (Sigma, Italy). A suitable amount of Milli-Q water (830 μL) and Folin-Ciocalteau reagent (50 μL) was added to 20 μL of the sample. After 3 min 100 μL of 6 % NaOH were added and the absorbance at 725 nm was measured after 1 h of incubation. The total phenol content was calculated by building a calibration curve with catechol as a reference. All analyses were carried out in triplicate.

### 2.4 HPLC analysis

The HPLC analysis of phenolic extracts was performed with an Agilent® 1100 instrument equipped with a pump and a diode-array detector. A Phenomenex 250 × 4.6 mm C-18 column with 4 μm particle size and a Phenomenex C-18 (4.6 × 30 mm) guard column were used. Detection was carried out at 279 nm. Elution gradients at a flow of 0.5 mL min^−1^ with a mobile phase composed of water acidified with *o*-phosphoric acid (solvent A) and acetonitrile-water (70:30 v/v) (solvent B) were adopted. The elution program for the OMW extract was as follows: isocratic elution with 85 % A for 5 min; gradient to 50 % A in 35 min; to 100 % B in 10 min; to 85 % A in 10 min; isocratic elution with 85 % A for 5 min. Standard solutions were necessary for the identification of the single phenolic molecules. Direct standard elution was used to ensure the identity of the compounds.

### 2.5 Catechol adsorption

Experiments of catechol (Sigma, Italy) adsorption by BP were performed to assess its capacity to adsorb phenols from OMW. Glass tubes (10 mL) were used to incubate 5 %, 10 %, and 15 % BP, with 1 mL of catechol solution at three different concentration (0.5, 1, and 1.5 mg mL^−1^). After 0.5 h, 24 h, and 7 days of incubation, the mixtures were centrifuged at 14000 rpm for 15 min and filtered (0.45 μm, Phenomenex). Filtrates were analyzed by HPLC as described by Kasozi et al. [20].

### 2.6 Biochar based treatment of OMW

BP and BC (5 and 10 %) were added to 40 mL of settled (s-OMW) or non-settled OMW. Notably, settled OMW were obtained leaving OMW to settle for 1 day. After that, the sediment was removed and only supernatant was used for biochar treatment.

After 20 and 60 days-biochar treatment phenolic content of OMW was evaluated and after 60 days germination tests (see below) was also performed to assess their phytotoxicity.

### 2.7 Soil amendment with biochar-treated OMW

Since the OMW treatment with BP induced a greater reduction of phenolic compounds compared with BC, soil amendment experiments were performed only with OMW treated for 60 days with BP. The detailed experimental design is described in Table 1 (Fig. S2).

**Table 1:** Experimental design of lab-scale soil amendment.

Sample	Composition
S	Control soil
BP5	Soil +5 % poplar biochar
BP10	Soil +10 % poplar biochar
OMW	Soil + OMW
s-OMW	Soil + OMW settled for 24h
OMW + BP5	Soil + OMW treated with 5 % poplar biochar
OMW + BP10	Soil + OMW treated with 10 % poplar biochar
s-OMW + BP5	Soil + OMW settled and treated with 5 % poplar biochar
s-OMW + BP10	Soil + OMW settled and treated with 10 % poplar biochar

The soil was placed in PVC tubes (30 cm × 5 cm) closed on the bottom with non-tissue disk. Tubes were stored at 24 °C and soil moisture was maintained at 30 % of the WHC by adding distilled water during the experiment. The OMW disposal occurred at 80 m^3^ ha^−1^ year^−1^ dose according to the Italian Legislative Decree 152/2006. After 30 and 90 days, chemical and biochemical analyses and seed germination tests of amended soil were carried out.

### 2.8 Chemical and biochemical analysis of soil

Soils amended with treated OMW and related control samples (without OMW and BP, see Table 1) were characterized and pH, electrical conductivity (EC), C, N, and available P were determined [33].

As regards biochemical properties, microbial C biomass (MB-C) was measured as soon as possible from the end of the incubation by fumigation-extraction method [17,39,40]. Enzyme activities were determined within 15–20 days from the soil sample collection. β-glucosidase (GLU) was determined as described by Eivazi and Tabatabai [41] and phosphatase (PHO) was determined according to Paredes et al. [42]. Urease (UR) activity was measured according to Kandeler and Gerber [43] whereas dehydrogenase (DH) activity according to Alef and Nannipieri [44]. The activity of the *o*-diphenol oxidase (DPO) was determined using a mixture of catechol and proline as the substrate [45]. The fluorescein diacetate hydrolase (FDAH) activity was assessed as described by Roccotelli et al. [46]. Triplicates were analyzed for each activity assay.

### 2.9 Phytotoxicity of soil amended with biochar-treated OMW

The germination test was performed in the presence of OMW treated with biochar (BP or BC) after 60 days and soil amended with treated OMW [18,46,47]. Briefly, *Lepidium sativum* L. seeds were placed in 10 × 90 mm Petri dishes (10 seeds per dish), equipped with 5 mL of OMW or 10 g of soil and incubated for 72 h at 25 ± 2 °C in the dark in a climatic chamber. Control test was performed with distilled water (experiment with OMW) or untreated soil (experiment with soil). The end of the germination process was reached when the thickness of the primary root was >1 mm. The relative germination

RG = 100 (Gs/Gc),

the relative length

RL = 100 (Ls/Lc),

and the germination index

GI = 100 (Gs/Gc) (Ls/Lc)

were calculated for each treatment. Gs and Gc are the numbers of seeds germinated in the sample and control, respectively, and Ls and Lc are the length of root in the sample and control, respectively.

### 2.10 Statistical analysis

Statistical analysis was carried out using SPSS (Version 23.0). Data were tested for normal distribution using the Shapiro–Wilk test and Levene's test was performed to assess the homogeneity of variance. The significant differences between means at p < 0.05 were assessed according to the Duncan test.

Principal component analysis (PCA) was performed on the Pearson correlation matrix to evaluate differences among all soil chemical, biochemical properties, and phytotoxicity tests, at two different sampling times (30 and 90 days), and the two first principal components (PC 1 and PC 2) were chosen for this study. The PCA was performed by XLSTAT (2016).

## 3 Results

### 3.1 Properties of OMW

OMW analysis showed an acidic pH (4.4 ± 0.1) and a high conductivity of 6.6 ± 0.2 mS cm^−1^ (Table 2).

**Table 2:** Chemical properties of OMW.

Chemical properties	Value
pH	4.4 ± 0.1
EC (mS cm^−1^)	6.6 ± 0.2
Total P (mg L^−1^)	177.5 ± 9.0
Dry matter (%)	3.6 ± 0.2
Water content (%)	96.4 ± 0.2
Chlorides (mg L^−1^)	177.5 ± 9.0
COD (mg L^−1^)	76227.5 ± 3626.1
BOD (mg L^−1^)	1583.0 ± 144.0
Total phenols (mg mL^−1^)	1.35 ± 0.10

COD and BOD values were consistent with literature data and total phenols placed, however, at intermediate levels of the ranges reported in the literature (Table 2) [48,49]. Most phenols were gallic acid, caffeic acid, catechol, protocatechuic acid, 2-hydroxybenzoic acid, and 2,6-hydroxybenzoic acid (Fig. S3).

### 3.2 Biochar-based treatment of OMW

**Fig. 1:**
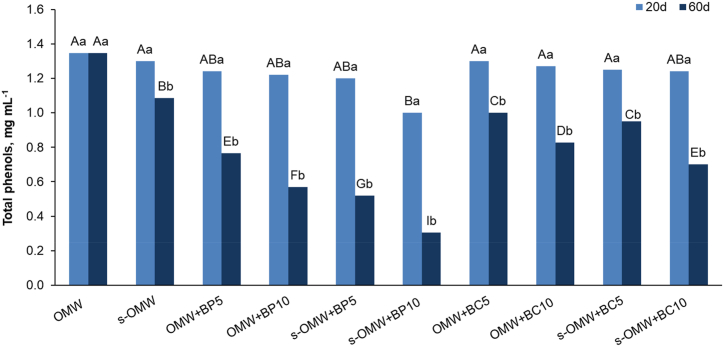
Total phenols in OMW differently treated with BP and BC after 20 and 60 days. Capital letters indicate significant differences between the treatments; lower case letters indicate differences between two incubation times (p < 0.05).

The treatments of OMW with BP and BC produced no significant reduction of phenolic content after 20 days. The total phenols of the treated OMW remained very close to 1.35 mg mL^−1^ (control sample) (Table 2 and Fig. 1) except for s-OMW treated with 10 % BP where the value significantly decreased up to 1.00 mg mL^−1^ (Fig. 1).

After 60 days the phenolic content in OMW control remained unchanged whereas it fell to 1.1 mg mL^−1^ in settled OMW (s-OMW; Fig. 1). At the same incubation time, biochar-based treatment (BP or BC) markedly reduced total phenols in both settled and not-settled OMW, although BP better performed in phenol reduction than BC. The samples OMW + BC10 and s-OMW + BC10 showed that the total phenol content reduced up to 61 and 65 % of the control value (0.83 and 0.70 mg mL^−1^ in OMW and s-OMW, respectively). Conversely, the treatment with 5 and 10 % BP highlighted a different response in OMW and s-OMW. After 60 days of incubation, the values of total phenols decreased by increasing the BP rate and by the settling process. Non-settled OMW contained only 57 and 42 % of the total phenols of the control upon 5 and 10 % BP treatment, respectively, whereas s-OMW registered 48 and 28 % of phenols after analogous treatment (Fig. 1).

To highlight the fate of phenols in OMW upon the biochar addition, the catechol adsorption by BP was carried out by preparing a catechol solution at increasing concentrations in a range similar to the total phenols’ concentration in OMW. No catechol polymerization occurred within 7 d as the solution remained colourless, however the removal of catechol molecules occurred in the presence of BP. The process depended on the catechol and biochar concentration as well as incubation time. In fact, in the sample Cat 0.5+BP5 0.4 mg mL^−1^ of catechol was removed after 30 min, thus reaching 80 % of the amount initially present in solution (0.5 mg mL^−1^). In contrast, catechol was completely removed by increasing the BP concentration to 10 and 15 % (Cat 0.5+BP10 and Cat 0.5+BP15) (Fig. S4a). By increasing the catechol concentration up to 1 and 1.5 mg mL^−1^, catechol disappeared after 24 h and 7 days, respectively (Figs. S4b and S4c).

### 3.3 Phytotoxicity of biochar-treated OMW

The germination test of *Lepidium sativum* seeds carried out in the presence of treated OMW and s-OMW confirmed the relationship between total phenols and phytotoxicity (Table 3). The treatment with BP10 removed the inhibition observed with untreated s-OMW, thus restoring completely RG (100 %). A partial removal of inhibition occurred with BP5 (RG 80 %). The RG values of not settled OMW remained unchanged with BP5 (0 %) whereas they rose to 60 % by increasing the BP rate (BP10; Table 3). The response of the root length (RL) to the presence of OMW treated with biochar was not as marked as that of RG. The RL values and as a consequence the GI values increased after the treatment but remained rather small (≤40 %) also with BP-treated OMW and s-OMW (Table 3).

**Table 3:** Phytotoxicity test of BP-treated OMW and BP-treated s-OMW (60 d) with *Lepidium sativum* L. seeds.

Sample	RG	RL	GI
	------------------------ % -------------------------		
OMW	0 g	0 g	0 g
s-OMW	0 g	0 g	0 g
OMW + BP5	0 g	0 g	0 g
OMW + BP10	60 c	35 d	20 f
s-OMW + BP5	80 b	33 d	30 e
s-OMW + BP10	100 a	40 d	40 d
OMW + BC5	0 g	0 g	0 g
OMW + BC10	0 g	0 g	0 g
s-OMW + BC5	0 g	0 g	0 g
s-OMW + BC10	80 b	35 d	40 d

Different lower-case letters indicate significant differences among the different treatments in according to Duncan post-hoc test (p < 0.05).

A positive effect of BC was not registered with OMW whatever the biochar rate and with s-OMW at 5 % BC (s-OMW + BC5). A recovery of RG and RL was observed only in the sample s-OMW + BC10 (Table 3). Considering these results, the next experiments were carried out by using exclusively BP-based remediated OMW for soil amendment.

### 3.4 Response of soil chemical properties to biochar-treated OMW

The soil used to test the effect of the BP-treated OMW amendment was a sandy soil (Table S1) having an alkaline reaction (pH 7.9), 12.1 g kg^−1^ of organic carbon, 1.2 g kg^−1^ of total nitrogen, and 46.1 mg kg^−1^ of available phosphate. The value of EC (0.1 dS m^−1^) was indicative of non-saline soil.

Changes in chemical properties in soil amended with BP-treated OMW did not occur in terms of pH and EC as no significant differences were observed after 30 and 90 days from OMW disposal (Table S2).

The organic carbon (OC) content significantly increased 30 days after the amendment in all treated soil samples, compared to the control one (12.2 g kg^−1^). In particular, soils amended with OMW or s-OMW + BP5 or BP10 reached or exceeded 14.0 g kg^−1^ OC. After 90 days of incubation time the OC content of all amended soils returned to its initial value: no significant difference was observed compared to the control soil (Table 4).

**Table 4:** Chemical properties and enzymatic activities of soil amended with BP-treated OMW and BP-treated s-OMW after 30 and 90 days from the amendment.

Samples	OC	N	P	MB-C	DH	FDAH	UR	GLU	PHO	DPO
	g kg^−1^		mg kg^−1^		μg g^−1^ h^−1^			μmol g^−1^ h^−1^		
*30 days*										
S	12.2 Ca	1.4 Ba	48.7 Ba	60.0 Eb	0.5 Ba	13.8 Ca	21.5 Aa	0.3 Ba	1.3 Ca	9009.7 Da
BP5	13.0 Ba	1.4 Ba	51.2 Ba	97.0 DEb	0.6 Ba	14.9 Ca	20.9 Aa	0.3 Ba	1.7 Ba	9157.8 Da
BP10	13.3 Ba	1.4 Ba	60.5 Aa	155.7 CDb	0.8 Ba	18.4 Ca	23.5 Aa	0.3 Ba	1.7 Ba	9773.9 Ca
OMW	13.6 Ba	1.4 Ba	53.7 Ba	177.7 CDb	0.8 Ba	16.7 Cb	20.6 Aa	0.4 Ba	1.7 Ba	7644.4 Ea
s-OMW	13.6 Ba	1.4 Ba	48.5 Ba	183.6 CDb	0.6 Ba	18.2 Ca	19.4 Aa	0.4 Ba	1.6 Ba	8079.2 Ea
OMW + BP5	14.2 Aa	1.4 Ba	53.0 Ba	200.0 Cb	1.2 Aa	35.7 Ba	18.0 Ab	0.4 Ba	1.9 Aa	7891.6 Ea
OMW + BP10	14.7 Aa	1.5 Aa	63.7 Aa	234.1 Bb	1.1 Aa	43.0 Aa	18.0 Ab	0.5 Aa	2.2 Aa	11578.4 Aa
s-OMW + BP5	14.0 Aa	1.4 Ba	50.0 Ba	201.7 Cb	1.0 Aa	36.0 Ba	19.4 Aa	0.4 Ba	1.8 Aa	10393.8 Ba
s-OMW + BP10	14.2 Aa	1.4 Ba	60.8 Aa	267.5 Ab	1.2 Aa	33.5 Ba	21.7 Aa	0.4 Ba	2.0 Aa	11062.1 Aa
*90 days*										
S	12.2 Aa	1.3 Bb	48.7 Aa	104.8 Fa	0.5 Ba	15.7 Ba	17.9 Bb	0.2 Bb	0.8 Bb	7005.6 CDb
BP5	12.1 Ab	1.4 Aa	52.0 Aa	187.4 Ea	0.4 Bb	17.3 Ba	20.4 Ba	0.1 Bb	0.9 Bb	7691.8 Cb
BP10	12.1 Ab	1.5 Aa	54.6 Ab	372.5 Ba	0.6 Ba	17.4 Ba	17.8 Bb	0.2 Bb	0.9 Bb	6850.0 Db
OMW	12.6 Ab	1.4 Aa	53.8 Aa	412.3 Aa	0.5 Bb	17.4 Ba	19.3 Ba	0.2 Bb	0.9 Bb	5713.3 Eb
s-OMW	12.5 Ab	1.4 Aa	49.6 Aa	352.0 Ba	0.6 Ba	14.9 Ba	20.4 Ba	0.2 Bb	1.0 Bb	6967.0 Cda
OMW + BP5	11.8 Ab	1.4 Aa	49.3 Ab	257.1 Da	1.0 Aa	20.5 Ab	24.8 Aa	0.4 Aa	1.8 Aa	6460.0 Db
OMW + BP10	12.1 Ab	1.5 Aa	52.4 Ab	310.5 Ca	1.0 Aa	22.3 Ab	27.0 Aa	0.4 Aa	2.0 Aa	9632.7 Ab
s-OMW + BP5	12.1 Ab	1.4 Aa	50.2 Aa	282.2 Da	1.0 Aa	20.4 Ab	20.2 Ba	0.4 Aa	1.8 Aa	8103.6 Bb
s-OMW + BP10	11.9 Ab	1.4 Aa	51.5 Ab	309.6 Ca	1.1 Aa	21.7 Ab	19.4 Ba	0.4 Aa	1.9 Aa	7903.2 Bb

OC: organic carbon; N: total nitrogen; P: Available phosphorous; MB-C: microbial biomass carbon; DH: dehydrogenase activity; FDAH: Fluorescein diacetate hydrolysis activity; UR: urease activity; GLU: β-glucosidase activity; PHO: phosphatase activity; DPO: *o*-diphenol oxidase. Different capital letters indicate significant differences among the different treatments in according to Duncan post-hoc test (p < 0.05). Different lower-case letters indicate significant differences between the two incubation times (30 and 90 days) in according to paired *t*-test (p < 0.05).

Nitrogen content changed slightly only after 90 days of incubation time in amended soils showing a significant increase (by 7–15 %) compared with the control soil (Table 4).

Besides BP10, the OMW + BP10 and s-OMW + BP10 enhanced markedly the available phosphorous content by 30.8 % and 24.8 %, respectively, after 30 days of incubation. This parameter fell to values close to that of control soil after 90 days in all soil samples (Table 4).

### 3.5 Response of soil biochemical properties to biochar-treated OMW

All amendments with biochar treated OMW stimulated the development of microbial biomass in terms of MB-C, which represents the fraction of organic carbon bound to soil microorganisms (Table 4). After 30 days a synergistic effect with the BP10-treated OMW and s-OMW occurred: the samples OMW + BP10 and s-OMW + BP10 reached 234.1 and 267.5 mg kg^−1^ MB-C, respectively, significantly greater values if compared to samples prepared with single components (BP 10, OMW or s-OMW; Table 4). Conversely, the amendment based on OMW + BP5 or s-OMW + BP5 produced no significant effect on soil microbial biomass compared to OMW and s-OMW. If BP10 alone affected positively this biochemical parameter (155.7 mg kg^−1^), BP5 did not increase it (Table 4).

After 90 days, the MB-C was still greater in many samples, and different response to different treatments was observed. The value of MB-C in OMW + BP5 and OMW + BP10 samples increased to 257.1 and 310.5 mg kg^−1^, respectively, although MB-C rose also in the control samples BP10, s-OMW, and, in particular, OMW that showed the biggest value (412.3 mg kg^−1^; Table 4).

Soil enzymatic activities, measured as bioindicators of soil fertility, gave a different response to differently treated OMW amendments (Table 4). The activity of DH, FDAH, and PHO significantly increased in soil 30 and 90 days after the amendment with OMW or s-OMW treated with BP5 and BP10 (i.e., OMW + BP5, s-OMW + BP5, OMW + BP10, s-OMW + BP10; Table 4). It is interesting to note that after 30 days the sample OMW + BP10 reached the highest value of the FDAH activity (43.0 μg g^−1^ h^−1^), three times greater than the control soil (S) (Table 4) and after 90 days the PHO activity decreased in all soil samples, but not in those amended with biochar-treated OMW (Table 4).

The urease activity (UR) (Table 4) in soils amended with OMW treated with BP5 or BP10 resulted not affected 30 days after the amendment. A positive response was registered after 90 days only in the samples OMW + BP5 and OMW + BP10 that reached 24.8 and 27.0 μg g^−1^ h^−1^, respectively (Table 4).

The activity of GLU showed, after 30 days, a slight significant increase only in the sample treated with OMW + BP10 (0.5 μmol pNP g^−1^ h^−1^) (Table 4). After 90 days, the GLU activity remained unchanged in all BP-treated OMW amended samples whereas its reduction was registered in S, BP5, BP10, OMW, and s-OMW (Table 4).

Compared to the control soil, OMW, s-OMW and OMW + BP5 showed a significant negative effect on DPO activity after 30 days; while this activity significantly increased in OMW + BP10, s-OMW + BP5 and s-OMW + BP10 samples (Table 4). After 90 days, all samples showed a significant decline in the DPO activity, maintaining however the same trend observed after 30 days.

### 3.6 Phytotoxicity of soil amended with biochar-treated OMW

Besides the chemical and biochemical properties of amended soil with differently treated OMW, the phytotoxicity of these soils was evaluated through the germination test of *Lepidium sativum* L. seeds.

As reported in Table 5, after 30 and 90 days from the beginning of the experiment, the amendment based on OMW, settled or not settled, treated with whatever biochar rate, did not affect germination parameters that, conversely, were significantly reduced by OMW or s-OMW. The settling process (s-OMW) enabled to contain the phytotoxic effect of this waste keeping higher the values of all indexes compared to OMW (Table 5). After 90 days from the amendment GI was 75 % for the OMW sample and 90 % for the s-OMW sample (Table 5).

**Table 5:** Relative germination (RG), relative length (RL) and germination index (GI) of *Lepidium sativum* L. seeds in the presence of soil amended with BP-treated OMW and BP-treated s-OMW after 30 and 90 days from the amendment.

Sample	RG	RL	GI
	------------------------ % -------------------------		
*30 days*			
S	100 a	100 a	100 a
BP5	100 a	100 a	100 a
BP10	100 a	100 a	100 a
OMW	80 b	75 c	75 c
s-OMW	90 b	80 b	80 b
OMW + BP5	100 a	90 b	100 a
OMW + BP10	100 a	100 a	100 a
s-OMW + BP5	100 a	100 a	100 a
s-OMW + BP10	100 a	100 a	100 a
*90 days*			
S	100 a	100 a	100 a
BP5	100 a	100 a	100 a
BP10	100 a	100 a	100 a
OMW	75 c	80 c	75 c
s-OMW	90 b	90 b	90 b
OMW + BP5	100 a	100 a	100 a
OMW + BP10	100 a	100 a	100 a
s-OMW + BP5	100 a	100 a	100 a
s-OMW + BP10	100 a	100 a	100 a

Different lower-case letters indicate significant differences among the different treatments in according to Duncan post-hoc test (p < 0.05).

### 3.7 Principal component analysis of the OMW amended soil

**Fig. 2:**
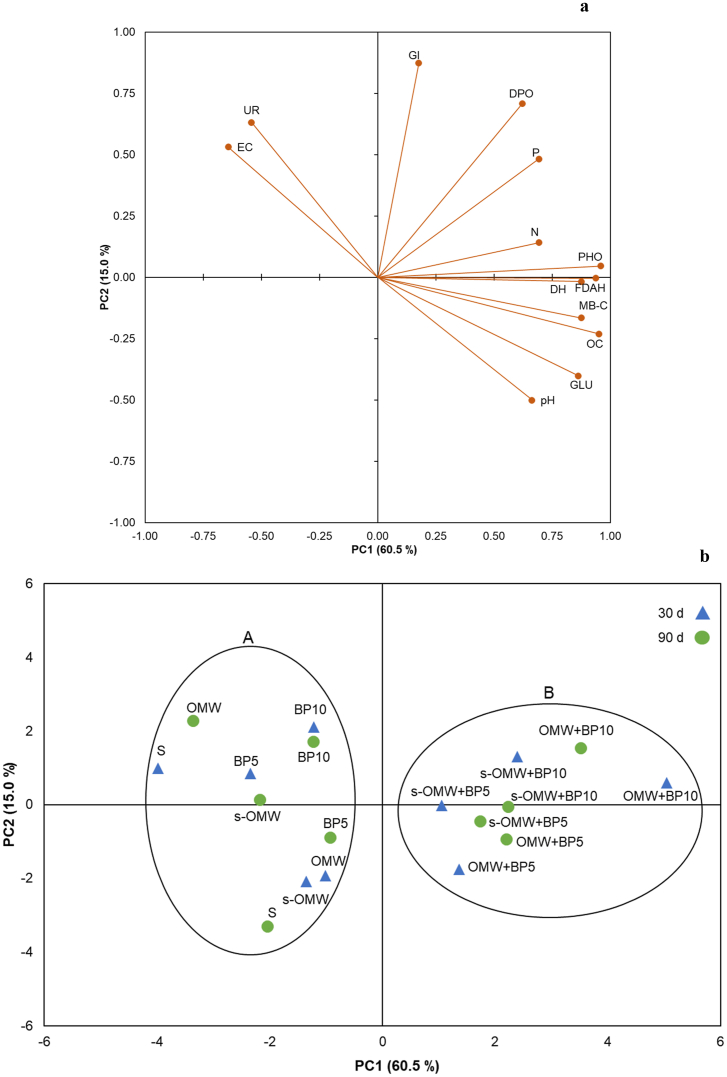
Principal component analysis of soil amended with BP or OMW and biochar treated OMW at two incubation times (30 and 90 days). a) Projection of the variables, b) projection of the cases.

Overall, to summarize the variability observed in chemical and biochemical properties and phytotoxicity test of the soil amended with untreated and biochar-treated OMW after 30 and 90 days, the discrimination of different treatments by Principal Component Analysis (PCA) was performed. The procedure allowed the extraction of two principal components (PC1 and PC2; Fig. 2). The two principal components, PC1 and PC2, accounted for 75.5 % of the whole variability. The PC1 (60.5 %) expressed the variability in terms of pH, OC, total N, available P, MB-C, DH, FDAH, GLU, and PHO activities (Fig. 2a). The PC2 (15.0 %) expressed the variability in terms of EC, GI, DPO, and UR activities (Fig. 2a). The PCA graphically discriminated soil samples in two clusters according to the amendment with untreated and biochar treated OMW along the PC1 (Fig. 2b). The first cluster (A), on the left, grouped the control sample (S) and the soil samples amended with BP5, BP10, OMW, and s-OMW at both incubation times. The second cluster (B), on the right, grouped soil amended with biochar-treated OMW (OMW or s-OMW + BP5 or s-OMW + BP10) at both incubation times.

## 4 Discussion

In our investigation, treating OMW with biochar considerably decreased the phenolic content, regardless of the origin biomass (poplar or conifer). The initial concentration of total phenolic compounds in OMW was 1.35 mg mL^−1^, within the typical range mentioned in the literature (up to 10 mg mL^−1^) [[50], [51], [52]]. The biochar treatment was efficient only after a long incubation time since a 20-day incubation time generated no changes in total phenols content compared to the value of the control sample (Fig. 1). After 60 days different responses to different treatments with BP and BC were observed. In comparison to the BC treatment, BP showed a greater reduction in phenolic compounds. The reason for this different effect was not consistent with the chemical structure and functional groups of biochars as FT-IR analysis did not highlight differences in the spectra of two biochars [22]. Instead, the physical properties of the two biochars were different: BP had a smaller pore volume and a smaller surface area than BC, as reported by De Pasquale et al. [32]. However, BP was characterized by a higher porosity due to a bigger number of small pores [32], a physical property that could favour phenol adsorption [20]. Better BP efficiency in OMW remediation was confirmed in our experiments (Fig. 1). Kasozi et al. [20] found that by increasing the pyrolysis temperature of biochar production catechol adsorption increased and the adsorption process took place in the micropores and nanopores. In addition, by increasing the biochar concentration an enhancement of phenol removal in OMW was observed. In our study, the pyrolysis temperature was the same for the production of both biochars (1200 °C), but in any case, the different observed porosity possibly resulted from the origin biomass [53]. In our previous study, BC showed a better performance than BP in the adsorption of phenanthrene, a polycyclic aromatic hydrocarbon, as in that case the access of big phenanthrene molecules was easier in the BC structure, having larger surface area and bigger pore dimension [22].

A germination test with OMW produced clear evidence of its phytotoxicity (Table 3) highlighting a negative correlation (r = −0.819) between phytotoxicity and phenol concentration. The greater reduction in phenol bioavailability for cress seeds obtained with BP led to a significant recovery of RG. Unfortunately, the detrimental effect on root length persisted. The RL values remained very far from 100 % of the control to indicate that the residual phenols could, however, negatively affect root elongation, especially in the first phase of growth, as demonstrated by other authors [1,2,7].

Despite OMW being handled differently and made less harmful, these findings may raise some concerns about the use of this waste in soil amendment. Nevertheless, it is important to take into account that this waste, after spreading into the soil, can undergo an inevitable dilution effect. In addition, not negligible issue, several processes may occur in the complex soil system such as OMW degradation as whatever organic material. Among these, oxidative polymerization of phenolic compounds can occur: biotic and abiotic catalysts such as soil enzymes (polyphenol oxidases, peroxidases) and Mn or Fe oxides, respectively, are responsible and polymeric particles are able to bind on/into soil particles (bound residues) [54,55]. All the process reduces OMW toxic potential [54,55]. In Italy, a specific law (Legislative Decree 152/2006) regulates OMW management and limits the disposal of this waste to 80 m^3^ ha^−1^ year^−1^, according to physical soil properties and the presence of groundwater to avoid leaching phenomena. A mitigated phytotoxic potential of OMW after biochar-based treatment could be a very interesting result to prevent in any case toxicity effect occurring in the first phase after the OMW disposal.

The use of biochar in detoxifying OMW allows for the disposal of the full mixture of OMW + biochar without first removing the biochar. Biochar is not poisonous or damaging to soil health rather it is useful and helpful in boosting soil fertility [56]. This could make easier and less expensive the application of this proposed methodology of OMW treatment.

Soil OC rose considerably after 30 days of amendment with BP5 and BP10, OMW and s-OMW, and even more with BP5- and BP10-treated OMW and -*s*-OMW. These findings might be explained by the action of biochar and OMW alone, but they are more likely to be explained by their synergistic effect. Indeed, Piotrowska et al. [57] proved the efficiency of OMW in improving soil organic matter thanks to its high COD. On the other hand, biochar could contribute, despite its recalcitrant nature, making available OC from its labile fraction [31].

A similar increasing effect occurred on soil available phosphorous only by BP10-treated OMW or s-OMW. BP10 alone can already enhance the available P, demonstrating that biochar may retain nutrients in its structure and act as a nutritional source [[58], [59], [60]].

Also MB-C, which represents the soil active and sporulated microbial biomass, benefited after 30 and 90 days from BP-treated OMW amendment, possibly due to the labile organic carbon provided by OMW and BP, but also from the improved soil physical properties and nutrient availability by BP contribution [31,56,59,61]. Moreno et al. [61] found that MB-C also significantly increased after 3 h from OMW spreading on soil. The enhancement of MB-C was positively correlated (p < 0.05) with the activity of DH (r = 0.85) and FDAH (r = 0.78) after 30 and 90 days of incubation time, according to numerous studies on the effect of organic amendments on soil biochemical properties [[62], [63], [64], [65]]. Indeed, the labile organic fraction from OMW stimulated, after 30 days, the activity of soil enzymes involved in C, N, and P cycles such as DH, FDAH, PHO, and DPO (Table 4). Conversely, GLU activity showed a positive response to the differently treated OMW only after 90 days from the amendment. The behaviour of UR is interesting because no significant impacts were detected regardless of treatment or incubation time. The ammonium component of OMW probably inhibits soil enzyme activity, therefore wiping out any favourable impact observed for other enzymes [57,61].

The increase of DPO activity, reflecting the phenol turnover in soil, in samples amended with BP-treated OMW was consistent with the lower phenol content of these latter [66]. By contrast, it was inhibited in samples with OMW and s-OMW, having higher phenol content.

As widely reported in the literature [2,[67], [68], [69]], OMW amended soil reduced seed germination, because of the OMW intrinsic characteristics [2]. As the biochar-based treatment of OMW decreased the phytotoxicity of this waste (Table 3), the germination of cress seeds and root elongation occurred properly as in the control sample (Table 5). However, OMW and s-OMW slightly inhibited both parameters (RG and RL) already after 30 days from wastewater disposal on soil. According to Tambone et al. [70] that consider wastewater not phytotoxic if the germination index is ≥ 60 %, also OMW and still better s-OMW seem not to affect the initial stage of plant growth at least at the maximum dose of 80 m^3^ ha^−1^ year^−1^ as regulated by the Italian law (Legislative Decree 152/2006). These findings allow hypothesizing and therefore advising that the regulated OMW quantities might be increased by using advantageous biochar treatment of OMW, hence enabling their disposal on agricultural soils.

The PCA graphically discriminated Cluster A containing the control soil and soil samples amended with only BP (5 or 10 %) or OMW or s-OMW from Cluster B containing soils amended with BP (5 or 10 %) treated OMW or s-OMW at both 30 and 90 days incubation (Fig. 2b). The greater PC1 scores for samples amended with biochar-treated OMW are a sign of the greater improvement of soil properties in terms of nutrient availability, microbial activity, and lack of phytotoxicity. On the contrary, all samples amended just with BP (5 or 10 %) or not treated OMW or s-OMW underwent no hard changes in terms of soil chemical and biochemical properties from control soil.

## 5 Conclusions

In general, biochar can be used in the treatment of OMW to reduce their phenolic compounds and phytotoxicity. The biochar-treated OMW disposal had a synergistic influence on chemical and biological soil characteristics. This positive effect was observed at both 30 and 90-day incubation times. The beneficial action of BP-treated OMW on soil properties is a clear sign of the loss of the polluting potential of this wastewater from olive oil mills. The suggested biochar based treatment might be a cost-effective solution to the long-term management of OMW: larger volumes could be spread out, reducing storage time, which is a key component of the process. Further investigations need to verify if a greater amount, also with repeated disposals, of biochar-treated OMW could further continue to improve soil properties. Field trials may be helpful for better understanding the real potential of biochar-based remediation of OMW.

## Authorship statement

The authors declare no competing financial interests or personal relationships that could influence this work.

## Data availability statement

Data will be made available on request.

## CRediT authorship contribution statement

**Giuseppe Di Rauso Simeone:** Writing – review & editing, Writing – original draft, Visualization, Validation, Supervision, Methodology, Investigation, Formal analysis, Data curation, Conceptualization. **Giuseppina Scala:** Methodology, Formal analysis. **Marcello Scarpato:** Methodology, Formal analysis. **Maria A. Rao:** Writing – review & editing, Visualization, Supervision, Resources, Project administration.

## Declaration of competing interest

The authors declare that they have no known competing financial interests or personal relationships that could have appeared to influence the work reported in this paper.
