# Supplementary material for: Response of chemical and biochemical soil properties to the spreading of biochar-based treated olive mill wastewater
Source: Heliyon. 2024 May 14;10(10):e31157. doi: 10.1016/j.heliyon.2024.e31157 (PMC11133665; doi:10.1016/j.heliyon.2024.e31157)
Supplement: Multimedia component 1 [file mmc1.docx]

**Supplementary Material**

**Biochar-based treatment of olive mill wastewater and effect of their spreading on chemical and biochemical soil properties**

Giuseppe Di Rauso Simeone, Giuseppina Scala, Marcello Scarpato, Maria A. Rao^*^

Department of Agriculture Sciences, University of Naples Federico II, via Università 100, 80055, Portici, Italy

*Corresponding author: Maria A. Rao e-mail: [mariarao@unina.it](mailto:mariarao@unina.it)

Pages: 8

Tables: 2

Figures: 3

**Content**

Table S1. Physical and chemical properties of soil.

Table S2. pH and Electrical conductivity of soil amended with BP-treated OMW and BP-treated s-OMW after 30 and 90 days from the amendment.

Figure S1. Experimental design from the OMW collecting to soil amendment.

Figure S2. PVC tube used in the lab-scale experiment.

Figure S3. Chromatogram of OMW at 279 nm. Numbers correspond to 1) gallic acid; 2) 2,6-hydroxybenzoic acid; 3) protocatechuic acid; 4) caffeic acid; 5) 2-hydroxybenzoic acid; 6) catechol; 7) syringic acid (internal standard).

Figure S4. Adsorption of a) 0.5 mg mL^-1^ , b) 1 mg mL^-1^ and c) 1.5 mg mL^-1^ catechol on BP 5%, 10% and 15%.

Table S1. Physical and chemical properties of soil.

| Properties | Value |
| --- | --- |
| Sand (g kg^-1^) | 879 ± 42 |
| Lime (g kg^-1^) | 93 ± 47 |
| Clay (g kg^-1^) | 27 ± 5 |
| pH (in H_2_O) | 7.90 ± 0.06 |
| EC (dS m^-1^) | 0.1 ± 0.03 |
| Limestone (g kg^-1^) | 6.2 ± 0.4 |
| CEC (cmol(+) kg^-1^) | 15 ± 1 |
| TOC (g kg^-1^) | 12 ± 1 |
| OM % | 2 ± 0.1 |
| Total N (g kg^-1^) | 1.22 ± 0.02 |
| C/N | 10 ± 0.7 |
| P_2_O_5_ (mg kg^-1^) | 46 ± 1 |

Table S2. pH and Electrical conductivity of soil amended with BP-treated OMW and BP-treated s-OMW after 30 and 90 days from the amendment.

| Samples | pH | EC |
| --- | --- | --- |
|  | - | dS m^-1^ |
| *30 days* |  |  |
| S | 7.22 Aa | 0.12 Aa |
| BP5 | 7.94 Aa | 0.12 Aa |
| BP10 | 7.91 Aa | 0.12 Aa |
| OMW | 7.9 Aa | 0.11 Aa |
| s-OMW | 7.85 Aa | 0.12 Aa |
| OMW+BP5 | 7.89 Aa | 0.12 Aa |
| OMW+BP10 | 7.86 Aa | 0.12 Aa |
| s-OMW+BP5 | 7.87 Aa | 0.12 Aa |
| s-OMW+BP10 | 7.88 Aa | 0.12 Aa |
| *90 days* |  |  |
| S | 7.87 Aa | 0.14 Aa |
| BP5 | 7.86 Aa | 0.14 Aa |
| BP10 | 7.69 Aa | 0.14 Aa |
| OMW | 7.68 Aa | 0.14 Aa |
| s-OMW | 7.72 Aa | 0.14 Aa |
| OMW+BP5 | 7.67 Aa | 0.14 Aa |
| OMW+BP10 | 7.65 Aa | 0.14 Aa |
| s-OMW+BP5 | 7.74 Aa | 0.14 Aa |
| s-OMW+BP10 | 7.73 Aa | 0.14 Aa |

Different capital letters indicate significant differences among the different treatments in according to Duncan post-hoc test (p < 0.05). Different lower-case letters indicate significant differences between the two incubation times (30 and 90 days) in according to paired t-test (p < 0.05).


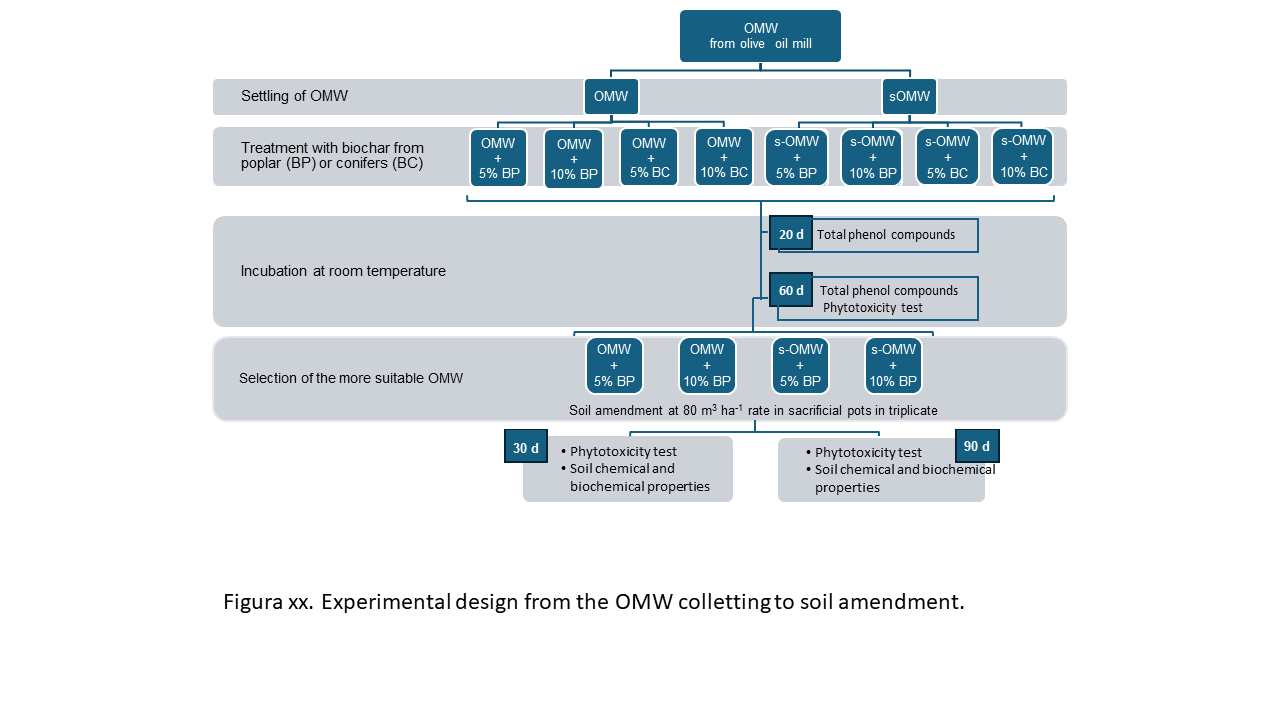


Figure S1. Experimental design from the OMW collecting to soil amendment.


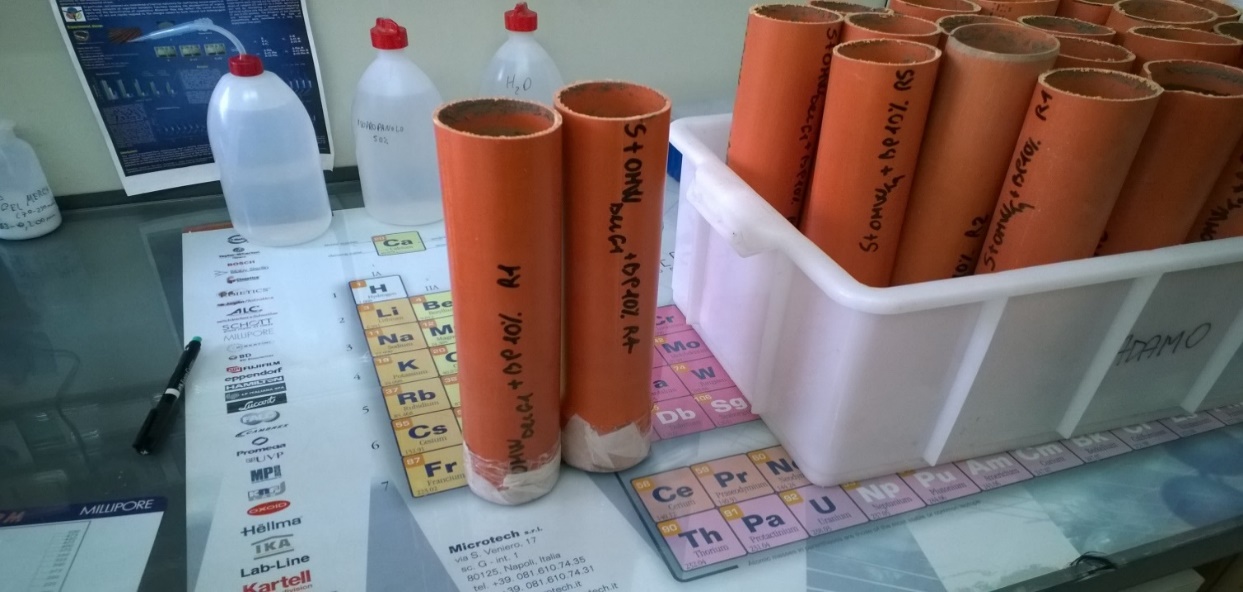


Figure S2. PVC tube used in the lab-scale experiment.


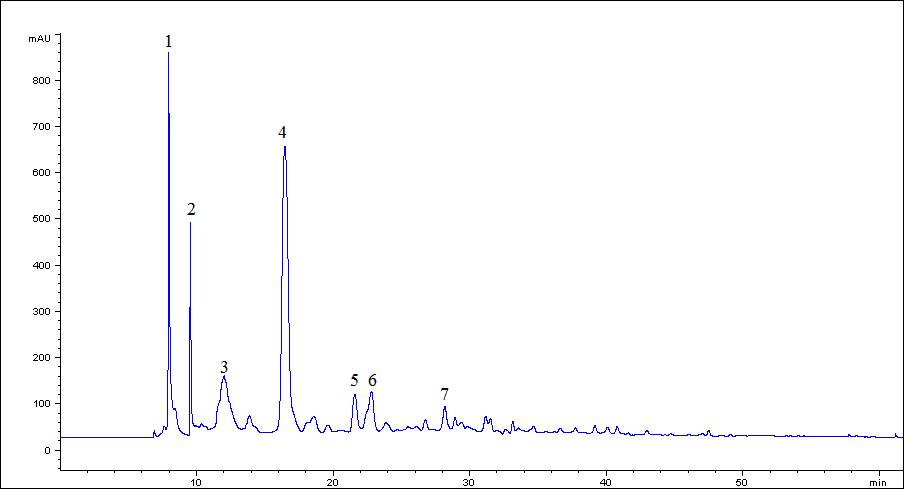


Figure S3. Chromatogram of OMW at 279 nm. Numbers correspond to 1) gallic acid; 2) 2,6-hydroxybenzoic acid; 3) protocatechuic acid; 4) caffeic acid; 5) 2-hydroxybenzoic acid; 6) catechol; 7) syringic acid (internal standard).


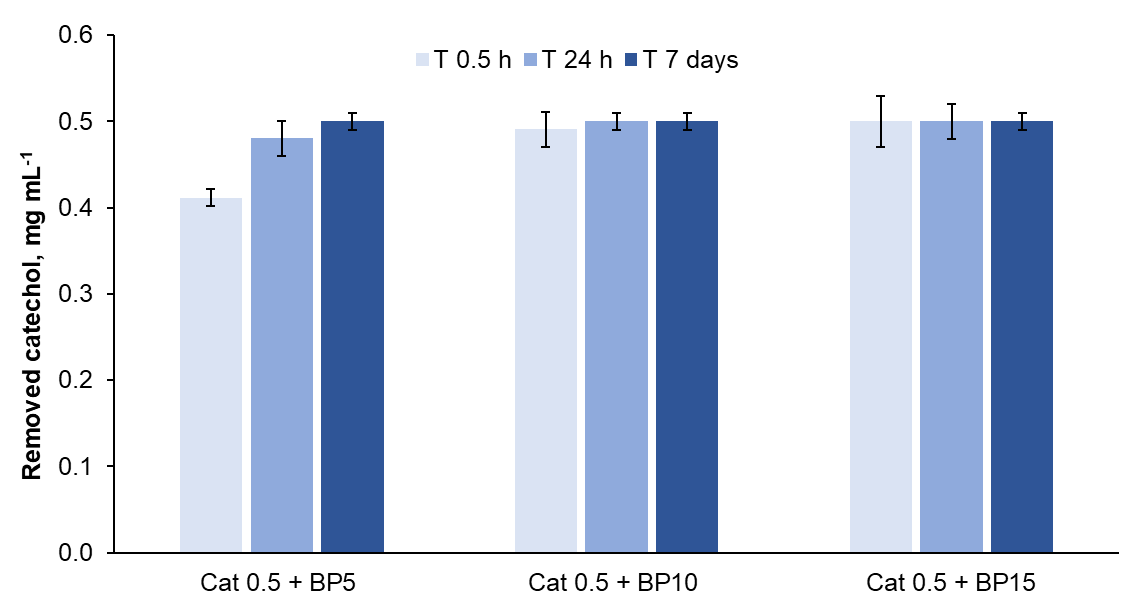


a


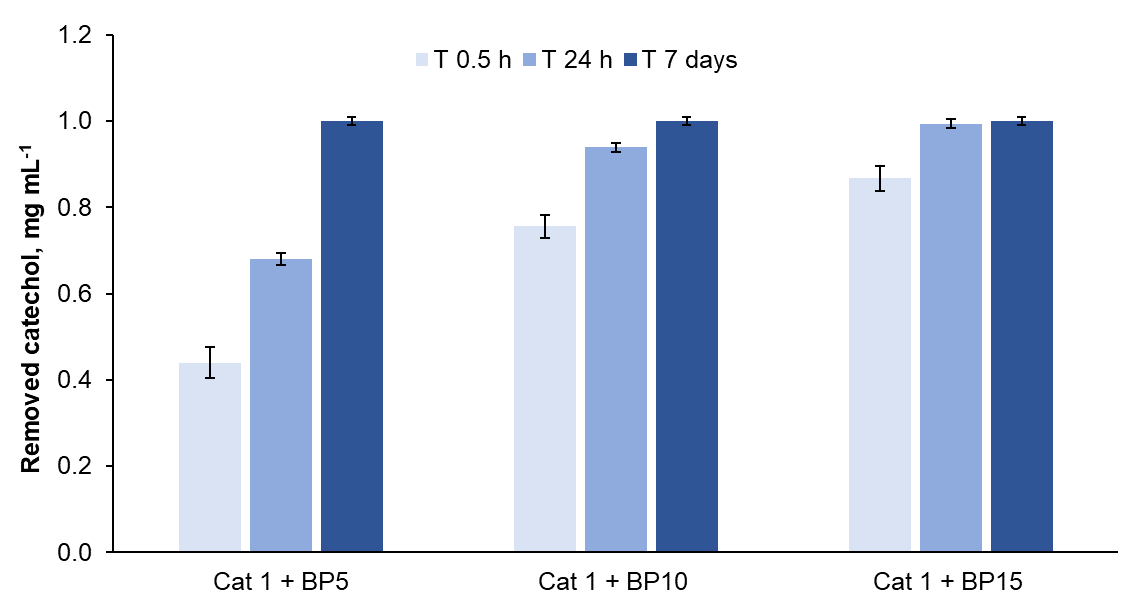


b


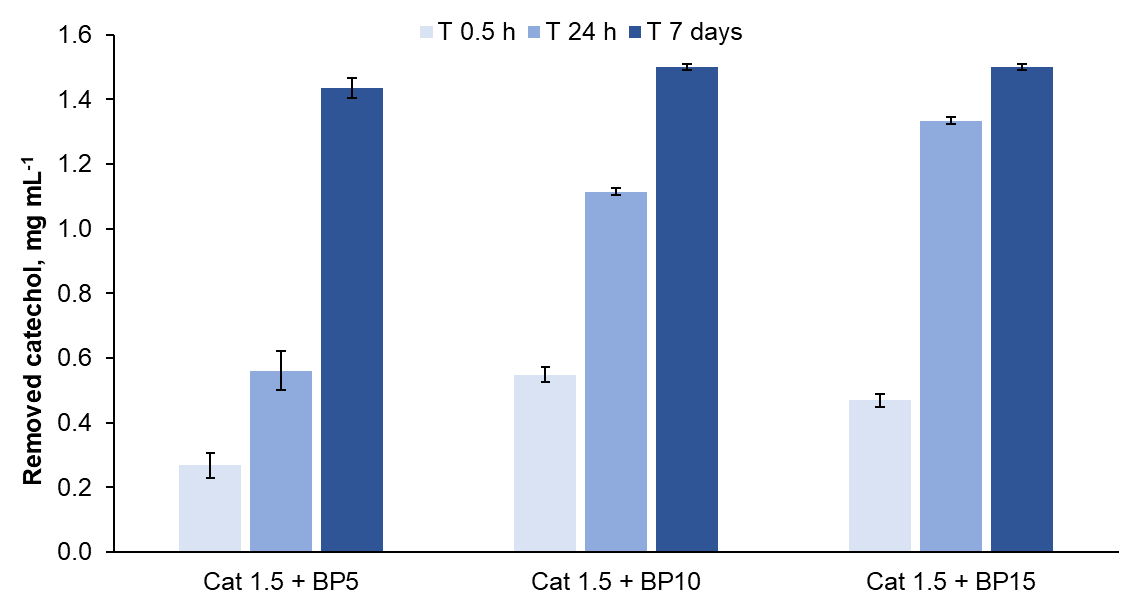


c

Figure S4. Removal of catechol in solutions prepared at 0.5 mg ml^-1^ (a), 1 mg ml^-1^ (b), and 1.5 mg ml^-1^ concentration by 5%, 10% and 15% BP.
